# Geographic location and ethnicity comprehensively influenced vitamin D status in college students: a cross-section study from China

**DOI:** 10.1186/s41043-023-00488-x

**Published:** 2023-12-20

**Authors:** Yingyi Luo, Chunbo Qu, Rui Zhang, Jingyi Zhang, Dan Han, Lixin Na

**Affiliations:** 1 Medical Technology College, Shanghai University of Medicine & Health Sciences, 279 Zhouzhu Road, Pudong New Area, Shanghai, 201318 China; 2 Public Health College, Shanghai University of Medicine & Health Sciences, 279 Zhouzhu Road, Pudong New Area, Shanghai, 201318 China

**Keywords:** Serum 25(OH)D_3_, Vitamin D deficiency, Latitude, Ethnicity, College students

## Abstract

**Background:**

Vitamin D plays an important role in the health of adolescents, whereas vitamin D status of Chinese college students was seldom studied in China. To explore the vitamin D status and its relationship with ethnicity and geographic location in Chinese college students.

**Methods:**

The freshmen were taken a physical examination by trained medical personnel after they reported to university. Demographic information including age, gender, ethnicity, region of original residence was collected using a questionnaire survey. Serum 25(OH)D_3_ concentrations were measured using a liquid chromatograph mass spectrometer. Multiple regression analyses were used to explore the factors that influence serum 25(OH)D_3_ levels.

**Results:**

Totally 3220 freshmen who came from 26 provinces, autonomous districts or municipalities were recruited, with a mean age of 18.75 ± 1.18 years and 70.2% of them were female. The mean serum 25(OH)D_3_ levels were 18.51 ± 6.54 ng/mL, and the proportion of vitamin D deficiency (< 20 ng/mL) and insufficiency (20 ~  < 30 ng/mL) was 64.4% and 30.2%, respectively. The combined proportion of vitamin D deficiency and insufficiency was increased with the latitude increased. Miao had the highest serum 25(OH)D_3_ levels, whereas Kazak ethnic had the lowest (22.51 ng/mL vs. 13.94 ng/mL) among different ethnic groups. Female students, students from city, Uighur and Kazak ethnic, residing in high latitude was significantly associated with lower serum 25(OH)D_3_ levels (*P* < 0.05).

**Conclusions:**

Vitamin D deficiency is an important health problem in Chinese college students. Sunlight activities, dietary and life-style intervention for college students according to geographic location and ethnicities should be considered.

## Background

Vitamin D plays an important role in promoting calcium and phosphorus metabolism and maintaining bone health. In recent years, many studies have also reported the association of vitamin D with non-skeletal diseases, including cardiovascular disease, metabolic syndrome (MetS) and cancer [1–4]. Therefore, vitamin D nutrition status and it influencing factors attract scientists’ attention regarding to nutritional prevention of disease.

In fact, vitamin D deficiency is still a major public health problem worldwide in all age groups [5–7]. Most studies about vitamin D status focused on children, pregnant and lactating women or old people, and the average deficiency rate of vitamin D in these populations was about 50–60% [8–11]. In China, the general prevalence of vitamin D deficiency in children and adolescents aged 6–17 was 53.2% at the cutoff of 50 nmol/L, according to the Chinese national nutrition and health survey 2010–2012 [12]. So far the reports about vitamin D nutrition status in youth were very limited, especially in college students in China. Recent studies have suggested that health conditions in early life including childhood and youth would influence risks of disease in middle and old age [13]. Therefore, it is also necessary to clarify whether vitamin D deficiency is a nutritional problem in youth from the point of view of disease early prevention using nutrition strategy.

Factors that affect vitamin D levels include age, gender, diet and ambient ultraviolet B levels, etc. [12]. Vitamin D is mainly produced in the skin by exposure to ultraviolet B in sunlight, and ultraviolet B levels vary in different latitudes, which may affect vitamin D levels in people [14]. Some studies have indicated high latitude was a statistically significant risk factor for vitamin D deficiency, and people at lower latitudes had higher levels of vitamin D [15, 16]. However, the sample size of these studies was small, and the coverage of latitude scope was limited. Large-scale studies across wide range of latitudes are still lacking. Ethnicity is another factor which is concerned to influence serum vitamin D levels [17, 18]. Therefore, it is necessary to comprehensively explore vitamin D status and its influencing factors in countries with a wide latitude scope and many ethnic groups for the formulation of national nutrition policy.

The purpose of this study is to explore the vitamin D status of the college freshmen who came from different provinces of China and to analyze the possible influencing factors of vitamin D levels especially latitude and ethnicity. This is critical for us to provide the theoretical supporting for the prevention of vitamin D deficiency.

## Methods

**Fig. 1:**
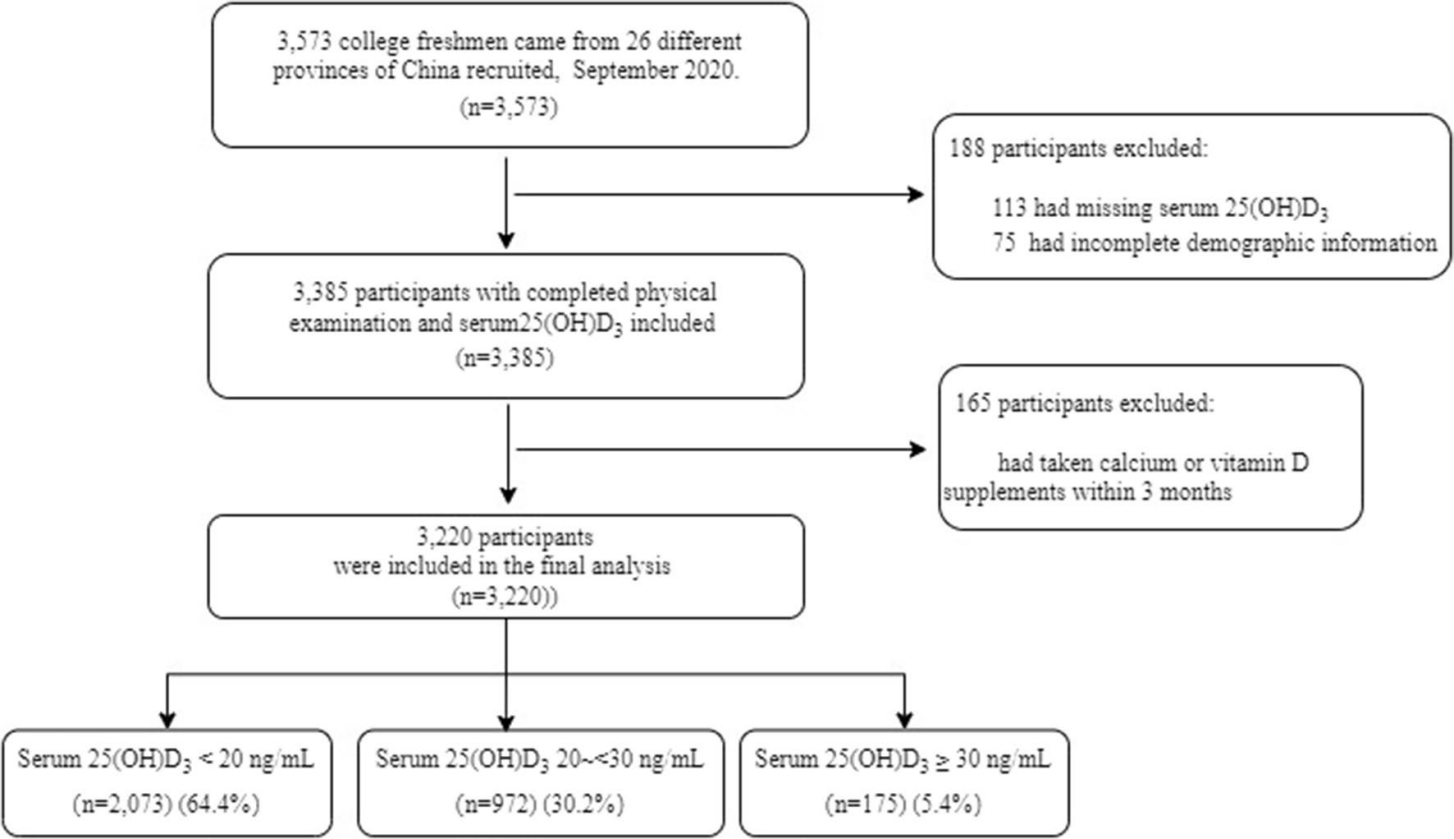
Flowchart for participants enrollment

The study subjects were freshmen of Shanghai University of Medicine and Health Sciences, started the school at 2020 September. They came from twenty-six different provinces of China and aged from sixteen to twenty-six. A total of 3573 students recruited into our study. After excluding 113 students who had missing serum 25(OH)D_3_ and 75 students who had incomplete demographic information, a total of 3385 students who had completed physical examination were included into our study. After excluding 165 students who had taken calcium or vitamin D supplements within 3 months, 3220 subjects were eventually included in our analysis. The flow of participants enrollment is shown in Fig. 1. This study was conducted following the guidelines outlined in the Declaration of Helsinki. All procedures involving human participants were approved by the Ethics Committee of Shanghai University of Medicine and Health Sciences. A written informed consent was obtained from all participants.

The freshmen were taken a physical examination by trained medical personnel after they reported to university. And a structured questionnaire was used to collect information on age, gender, ethnicity, region of original residence of all the participants.

Latitude and longitude were used to describe the location of students’ original residence. The information of latitude and longitude was obtained by checking the Chinese longitude and latitude Information Table after the region of original residence had been collected. According to the geographical scope of the subjects' original residence in our study, the latitude position was divided into four categories at a 10-degree interval:15° N–24° N, 25° N–34° N, 35° N–44° N, 45° N–54° N, and the longitude position was divided into three categories at a 20-degree interval: 75° E–94° E, 95° E–114° E, 115° E–134° E.

Fasting venous blood samples were collected and transported in ice boxes to Shanghai University of Medicine and Health Sciences for the assessment of serum 25(OH)D_3_ concentration. Serum 25(OH)D_3_ concentration was measured using a high-performance liquid chromatography (Agilent 1100; Agilent Technologies Inc., Santa Clara, CA, USA) and a mass spectrometer (API4000Q trap; AB SCIEX LLC., Redwood City, CA, USA). The lower limits of 25(OH)D_3_ for detection were 1.6 ng/mL. The test sensitivity was assessed with the inter-batch coefficient of variation (CV) of 5.85% and between batches CV of 6.18%.

According to the recommendation of Chinese Medical Association based on the Consensus of the Chinese Society of Osteoporosis and Bone Mineral Research, circulating 25(OH)D_3_ < 10 ng/mL was considered severely deficient, 10 ~  < 20 ng/mL deficient, 20 ~  < 30 ng/mL insufficient and ≥ 30 ng/mL sufficient [19].

SPSS 22.0 (IBM Corp., Armonk, NY, USA) was used for statistical analysis. The category variables were expressed as frequency (percentage) and numerical variables as mean ± standard deviation. The χ^2^ test was used to analyze the association between categorical variables, and Student’s t test or one-way analysis of variance (ANOVA) with the least squares differences (LSD) method was used to test differences among groups of continuous variables. Bootstrap test was used to test differences of serum 25(OH)D_3_ among the minorities in Xinjiang, Han in Xinjiang and Han in Shanghai because of the small sample size of Kazak and Han in Xinjiang. Multiple regression analysis was used to explore the factors that influence serum 25(OH)D_3_ level, with serum 25(OH)D_3_ levels as dependent variable and the independent variables included age, gender, student’s original residence, ethnicity, latitudes (as continuous variables) and longitude (as continuous variables). A two-sided *P* < 0.05 was considered statistically significant.

## Results

Basic characteristics with the serum 25(OH)D_3_ means of the participants are shown in Table 1. A total of 3220 participants with complete data were included in this study, including 960 males (29.8%) and 2260 females (70.2%). The participants came from twenty-six provinces, autonomous districts or municipalities of China with an age ranged from 16 to 26 years old, and the average age was 18.75 ± 1.18 years. Levels of serum 25(OH)D_3_ of the participants ranged from 5.20 to 64.83 ng/mL with the mean of 18.51 ± 6.54 ng/mL The proportion of vitamin D deficiency and insufficiency was 64.4% and 30.2%, respectively. The mean serum 25(OH)D_3_ of male was higher than female, and the mean serum 25(OH)D_3_ of participants who came from countryside and town were higher than those came from city. Participants came from high-latitude regions had relatively lower serum 25(OH)D_3_ levels, and participants came from central longitude had relatively higher serum 25(OH)D_3_ levels.

**Table 1:** Characteristics of the participants with serum 25(OH)D_3_ means and 95% confidence intervals (*n* = 3220)

Variable	*n*	%	Mean(ng/mL)	95%CI	*P* value
Age	–	–			
16–17	63	2.0	19.62	(17.94–21.30)	0.130
18–19	2697	83.8	18.42	(18.18–18.66)	
20–26	460	14.3	18.91	(18.27–19.55)	
Gender					
Male	960	29.8	21.41	(20.94–21.87)	< 0.001
Female	2260	70.2	17.28	(17.05–17.52)	
Region of original residence					
City	2017	62.6	17.83	(17.56–18.11)	< 0.001
Town	410	12.7	19.34	(18.68–19.99)	
Countryside	793	24.6	19.82	(19.35–20.28)	
Ethnicity					
The Han Ethnic	3050	94.7	18.52	(18.29–18.75)	0.767
The minorities	170	5.3	18.37	(17.31–19.43)	
Latitude					
15° N–24° N	108	3.4	21.45	(20.11–22.79)	< 0.001
25° N–34° N	2749	85.4	18.44	(18.19–18.68)	
35° N–44° N	331	10.3	18.31	(17.68–18.95)	
45° N–54° N	32	1.0	17.15	(15.03–19.28)	
Longitude					
75° E–94° E	56	1.7	15.66	(14.32–16.99)	< 0.001
95° E–114° E	570	17.7	20.34	(19.79–20.89)	
115° E–134° E	2594	80.6	18.17	(17.92–18.42)	

**Fig. 2:**
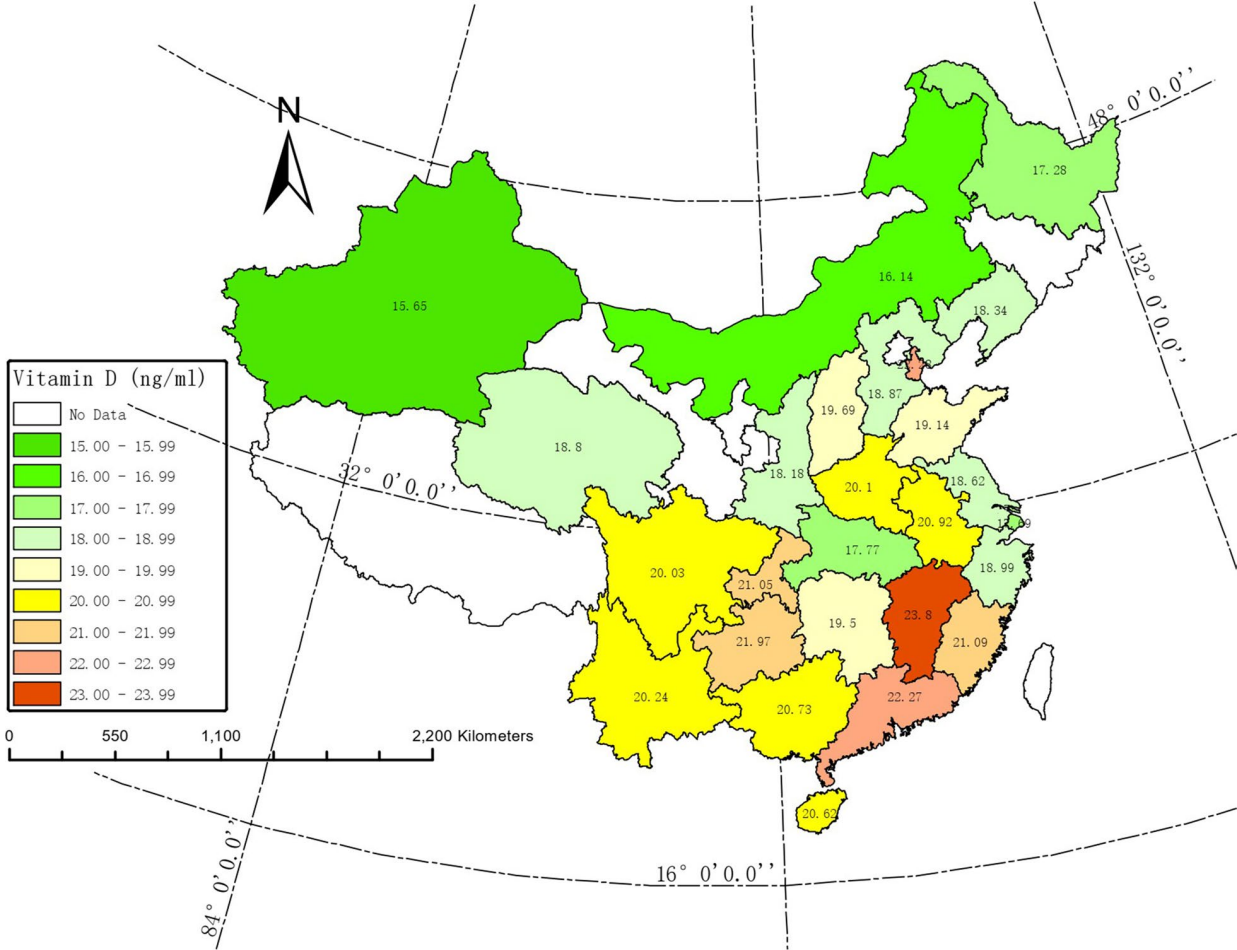
Map of China with Serum 25(OH)D_3_ means in ng/mL for participants from different Province (*n* = 3220)

The mean of serum 25(OH)D_3_ levels for participants who came from twenty-six different provinces of China was shown according to province, respectively, in Fig. 2. The mean of serum 25(OH)D_3_ levels ranged from 15.65 ng/mL (Xinjiang, latitude:34° 22′ N–49° 10′ N) to 23.80 ng/mL (Jiangxi, latitude: 24° 29′ N–30° 04′ N). According to the heat map of China we made, the mean of serum 25(OH)D_3_ levels in north was relatively lower than that in south, and it was also relatively lower in west than that in east.

**Fig. 3:**
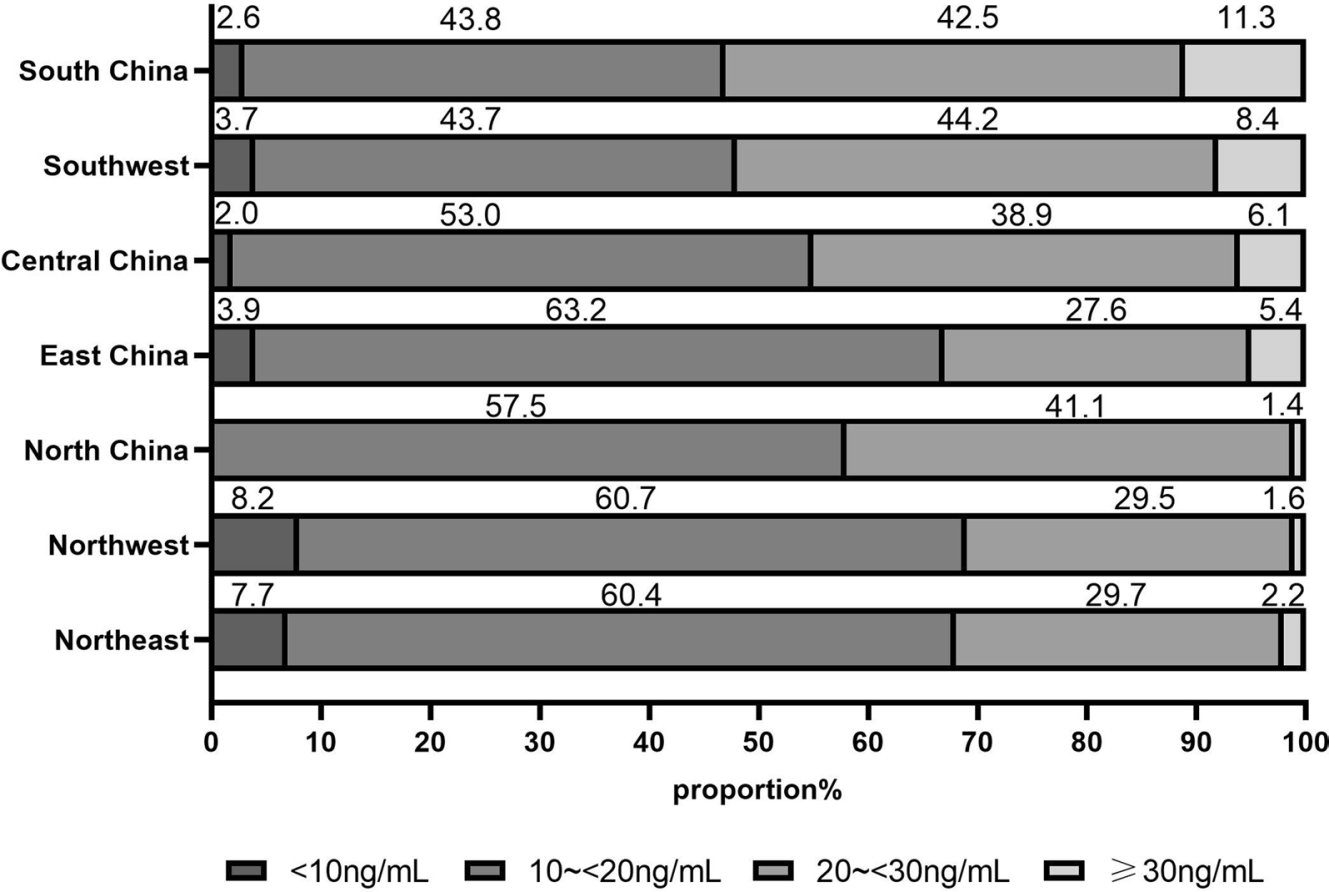
Proportion of Serum 25(OH)D_3_ categories by administrative areas in Chinese college students (*n* = 3220)

**Fig. 4:**
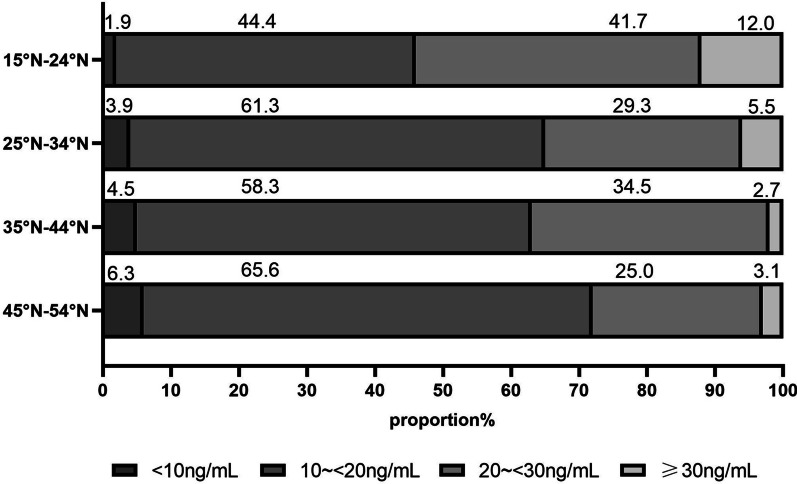
Proportion of Serum 25(OH)D_3_ categories by latitudes in Chinese college students (*n* = 3220)

The percentage of vitamin D deficiency and insufficiency in different areas of China is shown in Fig. 3. China is usually divided into 7 administrative areas based on the features of physical geography, and they are South China, Southwest, Central China, East China, North China, Northwest and Northeast. In Fig. 3, the proportion of vitamin D deficiency/insufficiency was the lowest in South China and Southwest (43.7%/42.5%–43.8%/44.2%) and the highest in North China, Northwest and Northeast (57.5%/29.5%–60.5%/41.1%). The highest sufficient rate was in south China, and it was only 11.3%. The percentage chart was also made according to the region latitude of the participants (Fig. 4). The combined proportion of vitamin D deficiency and insufficiency was increased with the latitude increased (88.5%, 94.5%, 97.3%, 96.9% for latitude 15° N–24° N, 25° N–34° N, 35° N–44° N, 45° N–54° N, respectively), and the percentage of vitamin D sufficiency was relatively lower in high latitude than that in low latitude.

**Fig. 5:**
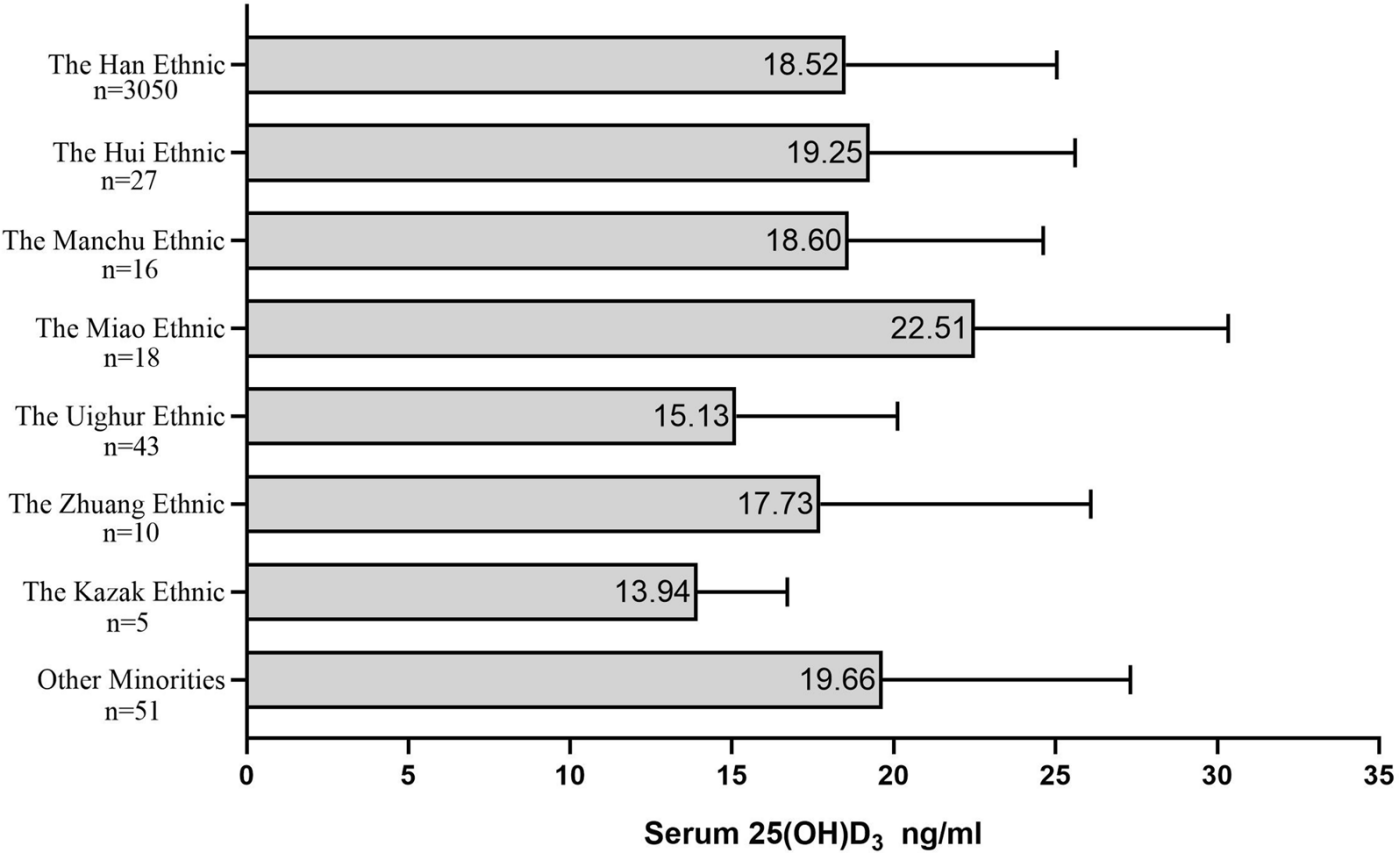
Mean levels of serum 25(OH)D_3_ for different ethnic of the participants (*n* = 3220)

**Fig. 6:**
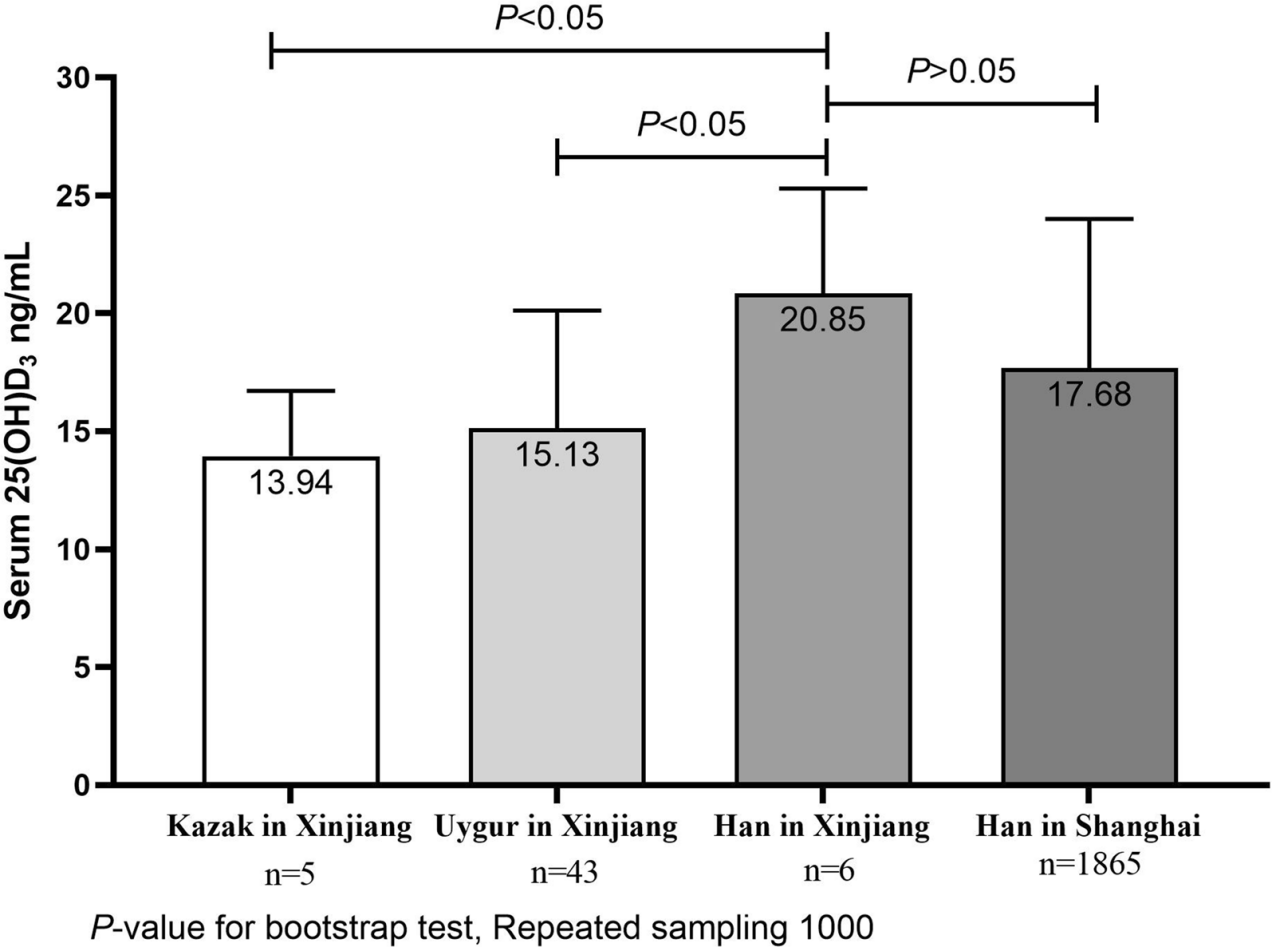
Mean levels of serum 25(OH)D_3_ for Kazak in Xinjiang, Uygur in Xinjiang, Han in Xinjiang and Han in Shanghai

The association of ethnicity with vitamin D levels is shown in Fig. 5. Participants with different ethnicities had different levels of serum 25(OH)D_3_ (*P* = 0.002). The participants of Miao had the highest serum 25(OH)D_3_ levels, whereas the participants of Kazak had the lowest (22.51 ng/mL vs. 13.94 ng/mL). In the present study, there were 3 ethnicities, including Kazak, Uighur and Han in Xinjiang. Further comparison of the serum vitamin D levels was made among Kazak in Xinjiang, Uighur in Xinjiang, Han in Xinjiang and Han in Shanghai (Fig. 6), so as to comprehensively analyze the influencing effects of ethnicity. Serum vitamin D levels were significantly lower in Kazak and Uighur than those in Han in Xinjiang by bootstrap test based on 1000 repeated sampling (Kazak: 13.94 ng/mL, Uighur: 15.13 ng/mL, Han in Xinjiang: 20.85 ng/mL, *P* < 0.05). There was no significant difference in Serum vitamin D levels between Han in Xinjiang and Han in Shanghai (*P* > 0.05) as shown in Fig. 6.

Multiple regression analysis showed that age, gender, region of original residence, ethnicity, latitude and longitude were significantly associated with serum 25(OH)D_3_ levels. Female students, students from city, Uighur and Kazak, tended to have lower serum 25(OH)D_3_ levels. Residing in high latitude was significantly associated with lower serum 25(OH)D_3_ levels after adjusted for longitude and other factors as shown in Table 2.

**Table 2:** Multivariate linear regression analysis of factors affecting vitamin D levels in Chinese college students (*n* = 3220)

Variable	*β* value	Standard *β* value	*P*-value
Age	0.279	0.050	0.003
Gender			
Male	–	–	–
Female	− 3.950	− 0.276	< 0.001
Students originally from			
City	–	–	–
Town	1.150	0.059	0.002
Countryside	1.744	0.115	< 0.001
Ethnicity			
The Han Ethnic	–	–	–
The Hui Ethnic	− 0.226	− 0.003	0.854
The Manchu Ethnic	− 0.138	− 0.001	0.930
The Miao Ethnic	1.782	0.020	0.227
The Uighur Ethnic	− 5.818	− 0.102	< 0.001
The Zhuang Ethnic	− 2.776	− 0.024	0.159
The Kazak Ethnic	− 5.824	− 0.035	0.045
Other minorities	− 0.552	− 0.011	0.535
Latitude (northern hemisphere)	− 0.114	− 0.060	0.001
Longitude (eastern hemisphere)	− 0.073	− 0.081	0.003

## Discussion

In this study, we tested the vitamin D status in 3320 college freshmen who came from 26 different provinces of China and explored the possible influencing factors of serum 25(OH)D_3_ levels. The mean serum 25(OH)D_3_ levels were 18.51 ng/mL, and the prevalence of vitamin D deficiency (< 20 ng/mL) and insufficiency (20 ~  < 30 ng/mL) was 64.4% and 30.2% respectively. Latitude and ethnicity were independent influencing factors of vitamin D levels in college students.

Studies have shown that vitamin D deficiency is still widespread around the world [5]. A study by Cashman et al. had estimated that the prevalence of vitamin D deficiency (< 50 nmol/L) was 40.4% for European population [20]. And Park JH demonstrated that the prevalence of vitamin D deficiency in 2008 was 51.8% in males and 68.2% in females, but rose to 75.2% and 82.5%, respectively, in 2014 in participants aged 10 years and older from the Korea National Health and Nutrition Examination Survey [21]. The study about the vitamin D status in college students was very limited. Nimri LF reported vitamin D deficiency reached 47.92%, and the mean 25(OH)D_3_ levels were 21.67 ng/mL among US female college students [22]. The mean serum 25(OH)D_3_ levels were 17.28 ng/mL (for female) in our study, which were much lower than the US college students and also lower than those in the elderly Chinese population (24.4 ng/mL) [23]. At the same time, the prevalence of vitamin D deficiency and insufficient was up to 94.6% by the cutoff of 30 ng/mL which was higher than the study of Jiang W in Chinese adult (83%) aged 18–65 from five different regions of China [24]. Our results indicated that vitamin D deficiency and insufficiency were serious in Chinese college students. The possible reason maybe that the freshmen had just experienced intense learning in high school and the outdoor activities were limited. Another possible reason maybe the higher proportion of girls in our study, and the vitamin D level of female was significantly lower than male which may lead to selection bias.

In this study, age, gender, region of original residence, ethnicity, latitude and longitude were significantly associated with serum 25(OH)D_3_ levels. Female was an independent factor of vitamin D deficiency in our study which was consistent with the previous study [25]. The relationship between latitude and vitamin D levels was a major focus of our study. Some studies in other countries had reported high latitude was a risk factor for vitamin D deficiency [15, 16]. In China, Jiang W reported the people from different regions had different serum vitamin D levels, but the sample came from only 6 key cities or provinces [24]. The participants of our study came from 26 different provinces of China, and we obtained the latitude and longitude information of their original residence region. The range of latitudes in our study was from 18 to 51° N. In our results, the mean serum 25(OH)D_3_ levels in northern were relatively lower than those in southern and the percentage of vitamin D deficiency or severe deficiency in high latitude was higher than that in low latitude. Therefore, people residing in high-latitude regions are key populations for primary and secondary prevention of vitamin D deficiency. Another phenomenon which we cannot ignore is that the percentage of vitamin D deficiency and insufficiency in low-latitude category (15° N–24° N) was still high to 82% by the cutoff of 30 ng/mL even in summer. The reason may be the college students in our study with the mean age of 18.75 ± 1.18 years have just gone through a period of rapid growth in life. They still need more amounts of vitamin D to boost calcium absorption. Another reason may be the college students especially female at this age in China usually pay more attention to sunlight protection, and they always wear sun-protective hats and sun-protective clothing and use sunscreen especially in summer. Another phenomenon in our study was that longitude was also significantly associated with serum 25(OH)D_3_ levels. Participants in central longitude (95° E–114° E) had higher serum 25(OH)D_3_ levels than those of West and East, this may be attributed to topography and climate of China. In a word, the vitamin D status of the college students in China was not optimistic. Adequate sunlight exposure may be one of the measures to prevent vitamin D deficiency in this age group.

Ethnicity is another factor which need to be concerned that affects vitamin D levels. Hsu S showed markers of vitamin D metabolism varied significantly by race/ethnicity, compared with Black participants, White participants had significantly higher serum 25(OH)D_3_ [26]. Study of Taksler GB has indicated that vitamin D insufficiency was pervasive among US minority populations, non-Hispanic Black race and Hispanic ethnicity were associated with a 7.47 ng/mL and 3.41 ng/mL decrease in vitamin D, respectively [27]. China is a multi-ethnic country, and ethnic groups included Han, Hui, Manchu, Miao, Zhuang, Kazak and other minorities in our study. Participants with different ethnicity had different levels of serum vitamin D. The participants of Miao had the highest serum vitamin D, whereas the minority of Uighur and Kazak had the lowest serum vitamin D in our study. This result was consistent with the study of Xu X that vitamin D insufficiency was highly prevalent in Uygurs and Kazaks living in Xinjiang, China [28]. In our study, the Uygurs and Kazaks are the distinctive ethnics in Xinjiang and they seldom live in other provinces of China. There were only three ethnics in Xinjiang province which were Uygurs, Kazaks and Han in our study. In order to further explore the relationship of vitamin D levels with different ethnic but in the same geographic location, we compared the vitamin D levels among Kazak, Uygur and Han in Xinjiang. We found the vitamin D levels of Kazak and Uygur in Xinjiang were significantly lower than Han in Xinjiang, whereas there was no significant difference in serum vitamin D levels between Han in Xinjiang and Han in Shanghai who had the similar gene and different geographic location. It suggested that genes may play an important role in affecting vitamin D levels among people in the same geographic location. The study of Xu X indicated that polymorphisms in CYP2R1-rs10766197 and DHCR7/NADSYN1-rs12785878 were associated with vitamin D deficiency in Uygur and Kazak ethnic populations ^(28)^, supporting a genetic effect on vitamin D status in minorities. Another reason maybe that the Kazak and Uygur people prefer to wear long trousers and long sleeves, as required by their culture. Therefore, influencing effects of lifestyles among different ethnic groups on vitamin D levels should also be concerned.

The strength of our study was that the participants of our study came from 26 different provinces which covered a wide geographical range of China. Taking college students as research subjects, we comprehensively compared the association of ethnicity and geographic location on vitamin D levels, thus filling the research blank of vitamin D deficiency in related fields of China. The limitation in our study was the participants of our study were freshmen of one school which cannot represent all college students in China. However, due to the wide geographical range of the study population, and the participants were taken a physical examination and were collected venous blood samples for the assessment of serum 25(OH)D_3_ as soon as they reported to university, the results can represent the vitamin D levels of Chinese college students to some extent.

## Conclusions

In conclusion, Chinese college students have relatively low levels of vitamin D, and vitamin D deficiency is an important health problem in Chinese college students. Latitude and ethnicity are independent influencing factors of vitamin D levels of college students. It is necessary to develop methods and strategies to improve the vitamin D status of college students. Sunlight activities should be encouraged to promote vitamin D synthesis among college students. Dietary and life-style intervention for college students according to geographic location and ethnicities should be considered.

## Data Availability

Data generated or analyzed during this study are included in this article and are available from the corresponding author on reasonable request.
